# Flexor Carpi Ulnaris Muscle Flap for Soft Tissue Reconstruction after Total Elbow Arthroplasty

**DOI:** 10.1155/2014/798506

**Published:** 2014-10-07

**Authors:** Syunro Okamoto, Kaoru Tada, Hachinota Ai, Hiroyuki Tsuchiya

**Affiliations:** Department of Orthopaedic Surgery, Graduate School of Medical Sciences, Kanazawa University, 13-1 Takaramachi, Kanazawa 920-8641, Japan

## Abstract

The soft tissue at the tip of the olecranon is very thin, leading to the frequent occurrence of wound complications after total elbow arthroplasty. To cover a soft tissue defect of the elbow, the flexor carpi ulnaris muscle flap is thought to be appropriate for reconstruction of the elbow with regard to its size, location, and blood supply. We got positive clinical results, so we report our experiences of using a flexor carpi ulnaris muscle flap for soft tissue reconstruction after total elbow arthroplasty.

## 1. Introduction

The soft tissue at the tip of the olecranon is very thin, leading to the frequent occurrence of wound complications after total elbow arthroplasty (TEA). Some methods were reported to cover a soft tissue defect of the elbow [1]. We think that the flexor carpi ulnaris (FCU) muscle flap is appropriate for reconstruction of the elbow with regard to its size, location, and blood supply. We report our experiences of using a flexor carpi ulnaris muscle flap for soft tissue reconstruction after TEA.

## 2. Case  1 

**Figure 1:**
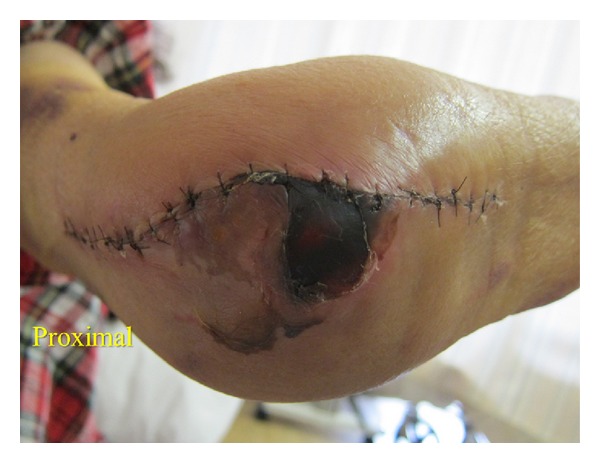
An area of skin necrosis occurred two weeks after surgery.

A 65-year-old woman with advanced rheumatoid arthritis, who takes 8 mg prednisolone daily, underwent TEA with a K-elbow prosthesis (BIOMET JAPAN Inc., Tokyo, Japan). Immediately after the operation, blistering occurred on the tip of the olecranon. An area of skin necrosis, 25 mm in circumference, occurred two weeks after surgery; a reconstruction using a FCU muscle flap was planned (Figure 1).

**Figure 2:**
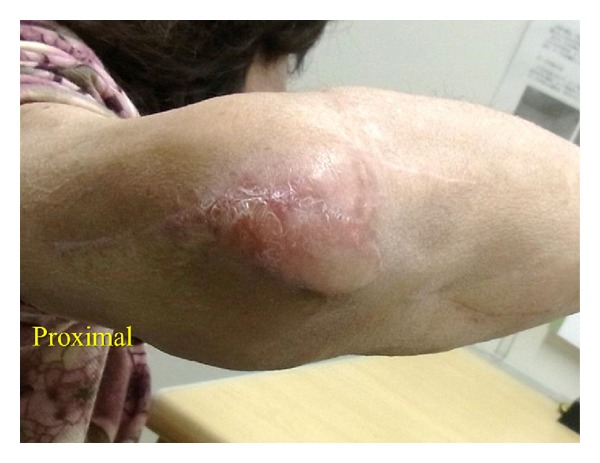
Nine months after reconstruction.

The operation was performed with the patient in the supine position. The FCU muscle was identified and cut at the level of the wrist joint. The incision was made directly above the FCU muscle with attention to preserve the medial antebrachial cutaneous nerve. After identifying a feeder artery branching from the ulnar artery in the proximal third of the forearm, we elevated and turned over the muscle flap to cover the olecranon. The FCU tendon was fixed to the surrounding tissues and the exposed muscle was covered with a full thickness skin graft. External fixation was carried out for two weeks after operation with decompressing flap. There were no postoperative complications. Nine months postoperatively, the range of motion in the elbow joint was 50 to 150 degrees (Figure 2). The Mayo clinic performance index for the elbow was 90/100.

## 3. Case  2 

**Figure 3:**
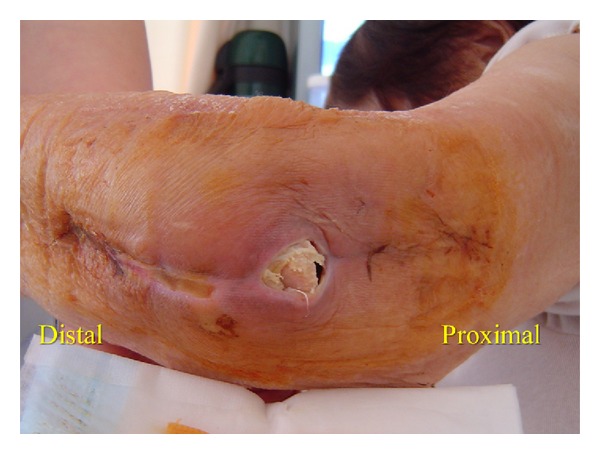
A wound dehiscence led to an exposure of the tip of the olecranon.

**Figure 4:**
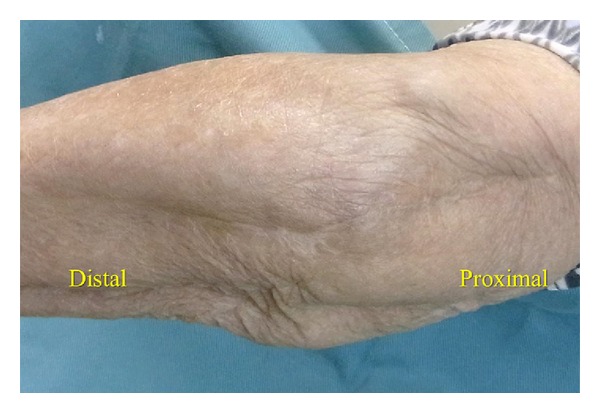
Three years after reconstruction.

An 84-year-old woman with advanced rheumatoid arthritis, who takes 8 mg prednisolone daily, underwent TEA with a Coonrad/Morrey total elbow prosthesis (Zimmer Inc., Warsaw, IN). Three weeks after surgery, she experienced a wound dehiscence and the tip of the olecranon was exposed (Figure 3). A reconstruction was indicated because of the poor skin condition around the wound, so we decided to use a FCU muscle flap. The operation was performed in the same manner as in Case 1. We were able to suture the wound with loose stiches rather than performing a skin graft over the FCU muscle flap. External fixation was carried out for two weeks after operation with decompressing flap the same as Case 1. There were no postoperative complications. At three years after surgery (Figure 4), the range of motion in the elbow joint was 35 to 130 degrees. The Mayo clinic performance index for the elbow was 95/100.

## 4. Discussion 

The tip of the olecranon has thin and movable skin, and postoperative complications after TEA are frequent. Jeon et al. report that 5–10% of TEA patients suffer postoperative wound complications: delayed healing, wound dehiscence, and necrosis [2]. Wound complications may expand to massive soft tissue defects; therefore, rapid treatment is paramount. The operative method must be selected according to the size of the soft tissue defect. We propose that defects less than 1 cm in width may be treated by an excision-and-suture method or a local flap, and defects over 1 cm must be treated by reconstruction using muscle or skin flaps. FCU [2, 3] and anconeus [4, 5] muscle flaps are commonly used. Latissimus dorsi myocutaneous flaps and free vascularized flaps are selected for massive defects.

The FCU muscle flap, including 2–4 cm of muscle belly, receives a blood supply from a branch of the ulnar artery in the proximal third of the forearm [6]. FCU muscle flaps may be rotated to cover defects on the tip of the olecranon [7, 8]; however, few instances of this procedure have been reported. We recommend a FCU muscle flap for the reconstruction after TEA because of the following 2 reasons. (1) It is easy to elevate the FCU flap. (2) A feeding artery of the FCU muscle flap is not disturbed after TEA. We propose that the FCU muscle flap is the most suitable choice for postoperative reconstruction after this procedure, especially for soft tissue defects smaller than 3 cm. We must, however, consider the functional disorders that may result in radial deviation and reduction in wrist flexor muscular strength. Lingaraj et al. used a split FCU muscle as a local muscle flap to avoid a functional loss at the wrist [9]. For patients with a thick muscle belly, this method may be an optional plan.

Another choice for reconstruction is the anconeus muscle flap. The anconeus muscle receives its blood supply mainly from the medial collateral artery and the recurrent posterior interosseous artery. Schmidt et al. [10] reported that the anconeus muscle flap can be expected to cover a defect over the olecranon if the muscle is harvested on the medial collateral artery. Though the anconeus muscle flap can be created close to the skin defect, limiting loss of elbow motion, we could not ignore the possibility of medial collateral artery injury, because the soft tissue of the distal humerus had been widely detached during the first operation. Morrey and Schneeberger advise that the blood supply of the anconeus muscle is uncertain in a patient with prior transection of the muscle's attachment at the triceps [11].

Jeon et al. recommend a reconstruction using a radial forearm flap, latissimus dorsi musculocutaneous flap, or free anterolateral thigh flap as the first choice for an intractable soft tissue defect, with the second choice being a FCU muscle flap. However, our opinion is that the least invasive treatment, the FCU muscle flap, should be selected first. This preserves the more invasive procedure, a latissimus dorsi musculocutaneous flap, for example, as a secondary choice or for salvage operations.
